# Comparison of Polynucleotide and Polydeoxyribonucleotide in Dermatology: Molecular Mechanisms and Clinical Perspectives

**DOI:** 10.3390/pharmaceutics17081024

**Published:** 2025-08-07

**Authors:** Sung Tae Kim

**Affiliations:** 1 Department of Pharmaceutical Engineering, Inje University, Gimhae 50834, Republic of Korea; stkim@inje.ac.kr; Tel.: +82-55-320-4038; 2 Department of Nanoscience and Engineering, Inje University, Gimhae 50834, Republic of Korea

**Keywords:** polynucleotide, polydeoxyribonucleotide, dermatological applications

## Abstract

Polynucleotide (PN) and polydeoxyribonucleotide (PDRN) are DNA-derived biopolymers increasingly recognized for their potential in dermatology. Despite their structural similarities, PN and PDRN exhibit distinct functions due to differences in polymer length and molecular weight. PN, composed of longer DNA fragments, plays a key role in extracellular matrix remodeling. Conversely, PDRN, composed of relatively shorter oligonucleotide sequences than those of PN, enhances skin condition through adenosine receptor activations and supports nucleotide synthesis via both the salvage and de novo pathways. This review provides a critical comparison of the molecular characteristics and functions of PN and PDRN with particular emphasis on their dermatological applications. By delineating their respective roles in esthetic and regenerative medicine, we aim to highlight recent advances that may guide the development of optimized treatment strategies and foster evidence-based clinical practice.

## 1. Introduction

The skin is the largest organ of the human body and performs a multitude of essential functions, including protection, temperature regulation, sensory perception, and immune defense [1,2]. It is composed of three main layers—the epidermis, dermis, and hypodermis—which work together to maintain physiological balance [3,4]. With advancing age, the skin undergoes several structural and functional changes, such as decreased collagen levels, reduced moisture content, lower sebum production, slower cell renewal, and accumulated damage from sun exposure. These changes impair the skin’s ability to maintain stability [5,6]. Such skin aging is influenced by both internal factors, such as hormonal and metabolic changes, and external factors, including ultraviolet (UV) radiation and environmental pollution [7]. Clinically, aged skin typically shows wrinkles, decreased elasticity, uneven pigmentation, and thinning. To address these issues, various esthetic and medical approaches have been developed to improve skin condition and enhance overall quality of life [8,9].

Polymeric nucleotide fragments, including polydeoxyribonucleotides (PDRNs) and polynucleotides (PNs), have recently emerged as promising agents [10,11,12]. The global polynucleotide market has been emerging and expanding rapidly [13], and a wide range of clinical applications are being actively implemented across dermatological and esthetic practices [14]. Both PN and PDRN are classified as deoxyribonucleotide-based polymers and have been predominantly used in clinic. However, PN and PDRN differ significantly in molecular chain length distribution, structural characteristics, and clinical applications. Their practical clinical applications vary depending on specific treatment objectives [15,16]. However, the distinct characteristics of PDRN and PN are substantially underrecognized, resulting in clinical misapplications and suboptimal treatment outcomes. Therefore, a clear understanding of the differences between these compounds is essential to ensure their appropriate and effective clinical application.

In this review, we aim to compare and analyze the differences between PDRN and PN from various perspectives, with a focus on skin health. We also discuss their properties and practical applications, spanning from bench research to clinical implementations, with particular emphasis on recent trends and future perspectives. Relevant literature was identified via searches of PubMed, Google, Google Scholar, and Web of Science, including English-language publications up to May 2025. Studies focusing on polynucleotides and polydeoxyribonucleotides were selected.

## 2. Chemical Structure of Polymeric Nucleotide Fragments

**Figure 1:**
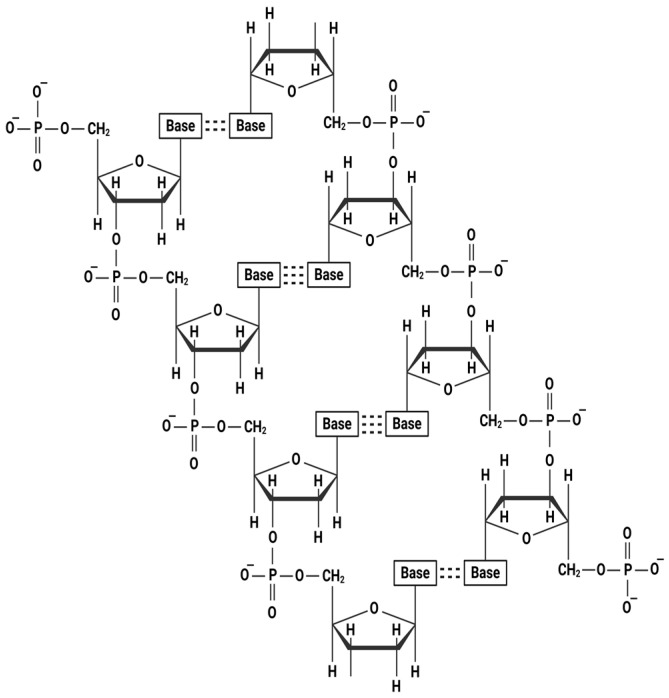
Basic chemical structure of polynucleotide PN and PDRN.

Both PN and PDRN are nucleic acid-derived polymers composed of nucleotide monomers connected via phosphodiester bonds [11,17]. Although PN broadly refers to covalently bonded nucleotide sequences, both PN and PDRN predominantly feature a comparable fundamental deoxyribonucleic acid (DNA) backbone as illustrated in Figure 1. However, they differ notably in molecular weight and polymer chain length. These molecules have attracted considerable attention since the 1990s because of their inherent biochemical and molecular biological relevance as genetic materials and functional biopolymers [15,18,19,20]. Previous studies have highlighted the considerable variation in fragment lengths between PN and PDRN [11,17]. PN, derived from the testes of fishes such as salmon or trout, typically consists of relatively longer chains [21,22]. On the other hand, PDRN is extracted from fish sperm and tends to consist of relatively shorter chains than those of PN. Furthermore, its sources mainly include the sperm of species such as *Oncorhynchus mykiss* (salmon trout) and *Oncorhynchus keta* (chum salmon) [11,19]. Recent studies have highlighted the ongoing nomenclature confusion between PN and PDRN, highlighting the need for clarification of their classification. PDRN, similar to PN, is composed of a linear structure of deoxyribonucleotides linked by phosphodiester bonds, which is double stranded [11,23]. However, PDRN exhibits lower viscoelasticity than that of PN because of its reduced molecular weight, whereas PN forms a more porous, highly viscous, three-dimensional structure [11,24,25]. These compositional and structural distinctions not only differentiate PN and PDRN at the molecular level but also suggest potential divergences in their biological behavior and mechanisms of action.

## 3. Biological and Physiological Role of PN and PDRN

### 3.1. Role and Function of PN

In dermatology, PN, primarily derived from fish testes, has shown promising benefits for skin health, including improving skin texture and elasticity, and reducing wrinkle depth [26,27,28]. From a regenerative perspective, PN contributes to creating a favorable microenvironment that supports extracellular matrix (ECM) production and overall tissue revitalization [29,30]. Owing to its high water content, PN forms a hydrogel that improves hydration and restores skin viscosity [31,32,33]. These properties have led to its application as both skin boosters and intra-articular injections [33,34]. Additionally, PN contributes to restoring the structural integrity and physiological function of the skin, and its effects are often amplified when used in combination with hyaluronic acid (HA) [35,36]. Because of its viscoelastic and hydrophilic properties, PN has been investigated as a potential alternative to HA in various applications [37]. Segreto et al. reported that the combination of HA and PN was demonstrated to be more effective than HA alone [38]. Dallari et al. and Stagni et al. also reported that a combination of HA and PN injections in a joint area was more effective than either agent alone [36,39]. Kim et al. demonstrated that the combined use of PN and HA promotes human fibroblast proliferation [40]. PN-HA treatments are well-tolerated and effective for repairing the skin barrier [33,35,41]. In addition, PN has shown favorable outcomes in improving knee function and alleviating pain without causing serious adverse effects [42,43,44,45,46,47]. Moreover, PNs create a favorable microenvironment for tissue rejuvenation and regeneration owing to their multifaceted clinical roles in promoting skin health.

### 3.2. Role and Function of PDRN

PDRN is a DNA-based therapeutic agent characterized by a relatively lower molecular weight than that of conventional PN. Structurally, PDRN consists exclusively of deoxyribose sugars in its backbone [48,49]. The pharmacological properties and clinical applications of PDRN have been extensively studied and are well-documented [50,51]. For instance, PDRN has been shown to stimulate the proliferation of skin fibroblasts [52] and osteoblasts [53], accelerate wound healing [25,54], promote angiogenesis [55], and exert notable anti-inflammatory effects [56]. These effects have been shown to be based on the activation of adenosine A2 receptors or degradation by the salvage pathway.

#### 3.2.1. Mode of Action of PDRN: Activation of Adenosine Receptor

Adenosine receptors belong to the purinergic G protein-coupled receptor family and mediate a wide range of biological and physiological effects of adenosine produced both intracellularly and extracellularly [57]. Adenosine, the endogenous ligand for adenosine receptors, exerts pleiotropic effects that vary depending on the cell type and tissue context. These include roles in neuromodulation [58], neuroprotection [59], long-term synaptic plasticity [60], vascular regulation [61], and immune modulation [62]. In addition to adenosine, several agonists (e.g., adenosine triphosphate), and antagonists (e.g., caffeine, theophylline), small molecules interact with adenosine receptors, resulting in diverse physiological outcomes [63,64,65]. Notably, the expression patterns and density of adenosine receptor subtypes differ across tissues and cell types.

**Figure 2:**
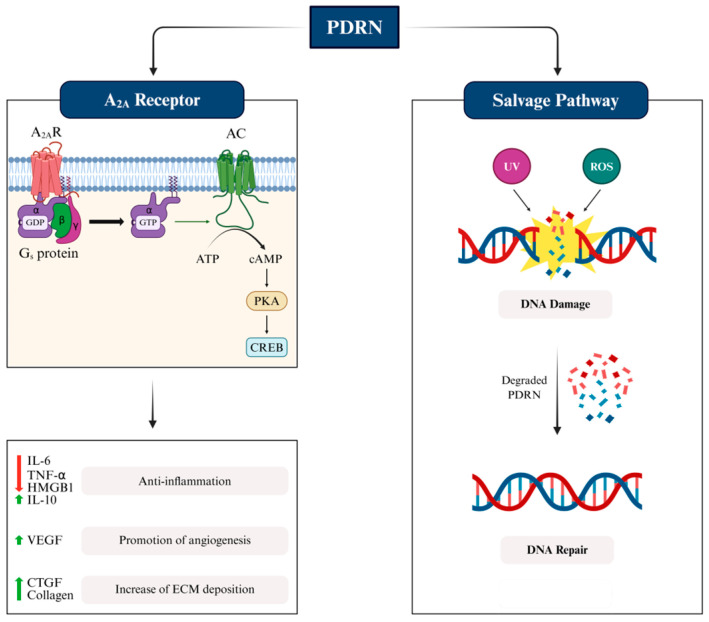
Mode of action of PDRN. Activation of adenosine A2 (A2A) receptor and the salvage pathway.

Among the four known subtypes of adenosine receptors—A1, A2A, A2B, and A3—PDRN selectively activates the A2A receptor. This effect modulates key physiological processes such as anti-inflammatory responses, angiogenesis, and vasodilation, as illustrated in Figure 2 (left panel) [52,66,67,68]. A2A receptor activation suppresses intracellular signaling pathways, including nuclear factor kappa B (NF-κB) and mitogen-activated protein kinase (MAPK) cascades, which inhibits proinflammatory responses and enhances anti-inflammatory mechanisms [69]. In addition, PDRN downregulates matrix metalloproteinase-1 (MMP-1), initiating the collagen synthesis cascade and enhancing collagen production in dermal fibroblasts [70]. PDRN acts as an adenosine receptor agonist to promote wound healing and tissue regeneration. These effects are primarily mediated through upregulation of vascular endothelial growth factor (VEGF) and interleukin (IL)-10, and downregulation of proinflammatory cytokines, including tumor necrosis factor-alpha (TNF-α), IL-6, high mobility group box-1 (HMGB1), and IL-1β [71,72]. Furthermore, PDRN enhances ECM deposition, especially collagen, by upregulating connective tissue growth factor. As shown in Figure 2 (the left panel), PDRN exerts its regenerative effects and accelerates wound healing primarily via activation of the A2A receptor.

#### 3.2.2. Mode of Action of PDRN: Degradation by Salvage Pathway

The nucleotide salvage pathway enables the efficient reutilization of bases and nucleosides released during the catabolism of DNA and RNA, in contrast to the energy-intensive de novo synthesis of purine and pyrimidine nucleotides [25,73,74]. Upon enzymatic degradation, PDRN yields a pool of bioavailable nucleosides and nucleotides that can be rapidly reincorporated into cellular processes, thereby promoting tissue regeneration and accelerating wound healing [50,75,76,77]. These molecular mechanisms become particularly critical in injured or hypoxic tissues, where de novo synthesis is compromised and the salvage pathway is the predominant source of nucleotide precursors for DNA synthesis [78]. While quiescent and differentiated cells preferentially utilize the salvage pathway, highly proliferative cells depend more heavily on nucleotide biosynthesis [79]. As illustrated in Figure 2 (right panel), PDRN-derived oligonucleotide fragments are sequentially hydrolyzed into nucleosides and nucleotides, which are subsequently recycled into the DNA of reparative cells. This incorporation restores the proliferative capacity and supports ongoing tissue repair [80]. In addition to fueling DNA synthesis, salvage-mediated recycling of nucleotides plays a pivotal role in maintaining genomic stability, particularly in dermal cells exposed to extrinsic insults (e.g., UV radiation) and intrinsic stressors (e.g., mitochondrial-derived reactive oxygen species (ROS)) [81,82]. By facilitating the efficient repair of DNA lesions, PDRN-derived nucleotides prevent activation of cell fate pathways associated with apoptosis or senescence, thereby preserving cellular function and tissue integrity [83,84].

### 3.3. Dermatological Effect of PDRN on Skin Health

#### 3.3.1. Skin Regeneration and Rejuvenation

Skin aging is driven by a combination of intrinsic and extrinsic factors that each contribute to distinct morphological and molecular changes [7,85]. Intrinsic aging is marked by epidermal thinning, progressive degradation of the ECM, and chronic low-grade inflammation driven by the accumulation of ROS [86]. In contrast, extrinsic aging, primarily attributed to UV exposure, manifests as photoaging, characterized by wrinkle formation, mottled pigmentation, and telangiectasia [86,87]. PDRN has shown significant rejuvenating effects on skin by exerting cell-specific actions through distinct molecular and signaling pathways [18,88]. In dermal fibroblasts, PDRN enhances metabolic activity and promotes the synthesis of essential ECM components—particularly collagen types I and III—through activation of the extracellular signal-regulated kinase (ERK) pathway [52,89,90,91,92,93,94]. Concurrently, it inhibits elastase and matrix metalloproteinase-1 (MMP-1), which are enzymes responsible for ECM degradation, preserving elastin integrity, improving skin elasticity, and reducing wrinkle depth [91,92,93]. Through these concerted actions, PDRN contributes to the restoration of ECM homeostasis, the repair of dermal architecture, and sustained skin rejuvenation. Moreover, by attenuating cellular senescence and enhancing dermal repair mechanisms, PDRN is an emerging and promising long-term therapeutic modality for improving and maintaining skin health.

#### 3.3.2. Skin Hypopigmentation

Melanocytes in the basal layer of the epidermis are responsible for pigmentation of both the skin and hair [95]. These cells synthesize melanin, a protective pigment primarily composed of eumelanin and pheomelanin, which is subsequently distributed throughout the epidermis [96]. Melanin plays a critical role in safeguarding the skin from extrinsic insults, such as UV radiation, and contributes to inter-individual variations in skin and hair color [97]. However, chronic UV exposure and inflammation can induce excessive melanin production and accumulation, leading to hyperpigmentation disorders such as melasma, freckles, mottled pigmentation, and senile lentigines [98,99]. Even in the absence of pathological melanogenesis, uneven or excessive pigmentation may pose esthetic concerns and adversely affect the psychosocial quality of life [100]. PDRN induces hypopigmentation by inhibiting melanogenesis through downregulation of melanogenic gene expression and suppression of tyrosinase activity, the rate-limiting enzyme in melanin synthesis [101]. Mechanistically, PDRN activates the ERK and phosphatidylinositol 3-kinase/protein kinase B (PI3K/AKT) signaling pathways, thereby suppressing microphthalmia-associated transcription factor (MITF), a master regulator of melanocyte function [102,103]. Although the precise molecular mechanisms remain to be fully elucidated, these findings position PDRN as a promising therapeutic agent for managing hyperpigmentation disorders. Further research is required to clarify these pathways and to optimize clinical protocols for the safe and effective application of PDRN.

#### 3.3.3. Skin Wound Healing

Skin injuries, including wounds and scars, arise from both intrinsic factors—such as impaired local blood supply—and extrinsic insults, including burns, trauma, and surgical interventions [104,105]. Constant exposure to such challenges causes the skin to sustain damage ranging from minor abrasions to extensive injuries that may culminate in fibrotic scar formation [106]. Breaches in skin integrity increase susceptibility to infections and often result in the development of non-functional, fibrotic scars [107,108]. Therefore, efficient wound healing, which restores the skin barrier and physiological homeostasis, is critical for protecting the body against environmental threats [109]. Wound healing is a complex and highly coordinated biological process involving cell migration and proliferation, ECM deposition and remodeling, inflammation, and angiogenesis [110]. PDRN has been demonstrated to enhance wound healing impaired by diverse pathological conditions, primarily via activation of the A2A receptor and engagement of the nucleotide salvage pathway, as previously described [111]. A2A receptor activation by PDRN reduces inflammatory cell infiltration, promotes proliferation and migration of reparative cells, stimulates vascular endothelial growth factor (VEGF) production, and facilitates fibroblast differentiation and maturation, thereby accelerating the reparative cascade [112,113]. A comprehensive review of in vitro and animal model studies and clinical trials shows evidence supporting the efficacy of PDRN in promoting wound closure and tissue regeneration [71,114]. These findings highlight the potential of PDRN as a promising alternative to existing bioactive agents, with significant therapeutic implications in wound management and regenerative medicine.

#### 3.3.4. Hair Regeneration

Hair serves essential functions including protection, sensory perception, and thermoregulation [115,116]. Hair loss, from aging, genetic predisposition, hormonal fluctuations, or pathological conditions, can lead to temporary or permanent thinning and baldness affecting the scalp and other body parts [117,118]. Consequently, promoting hair regrowth and regeneration remains a critical goal in clinical dermatology and esthetic medicine [119]. Although clinical data are limited, emerging evidence supports the therapeutic potential of PDRN in hair restoration. For example, co-treatment with platelet-rich plasma and PDRN has demonstrated improvements in hair thickness and density in patients with female-pattern hair loss [120]. Similarly, the use of PDRN alongside fractionated thulium laser therapy has shown promising results in enhancing both hair density and thickness [121,122]. These findings suggest that combinatorial approaches incorporating PDRN may effectively stimulate hair regrowth and improve overall hair quality and health.

#### 3.3.5. Anti-Inflammation in Skin

Inflammatory skin conditions, characterized by erythema, pruritus, pain, and dryness have diverse etiologies including environmental toxins, infections, injury, and autoimmune diseases [123,124]. These inflammatory responses involve complex interactions among immune cells, dermal fibroblasts, and smooth muscle cells, culminating in the release of proinflammatory cytokines and chemokines [125]. Although research on the dermatological effects of PDRN is limited, accumulating evidence indicates that PDRN downregulates proinflammatory cytokine expression across various in vitro and in vivo disease models [126]. In monocyte/macrophage cell lines such as RAW 264.7, PDRN attenuate inflammation by suppressing inflammatory mediator production [127]. Moreover, in an imiquimod-induced murine model of psoriasis—an archetypal chronic inflammatory skin disease—PDRN mediates anti-inflammatory effects through activation of the adenosine A2A receptor [128]. Similarly, PDRN inhibits proinflammatory responses in human dermal fibroblasts, further underscoring its immunomodulatory potential [129]. Collectively, these findings support the prospect of PDRN as a promising anti-inflammatory agent in dermatological applications. Future investigations should aim to clarify the detailed signaling pathways involved in PDRN-mediated anti-inflammatory effects and to evaluate its clinical efficacy across diverse inflammatory skin diseases.

#### 3.3.6. Antioxidant Effect on Skin

Oxidative stress is a key driver of skin aging induced by extrinsic factors such as UV radiation, air pollution, and smoke, and by intrinsic factors including cellular respiration, metabolism, and inflammation [130]. The interplay of these factors elevates ROS levels, disrupting redox homeostasis, and contributing to cellular senescence and various skin disorders [130,131]. This imbalance of excessive free radical generation and diminished antioxidant defenses accelerates skin aging and dysfunction [132,133]. Although these studies on the antioxidant properties of PDRN are limited, recent evidence demonstrates that PDRN alleviates oxidative stress in vitro, as shown in RAW264.7 macrophage injury models. These findings are supported by proteomic analyses and conventional antioxidant assays, including 2,2-diphenyl-1-picrylhydrazyl, 2,2′-azino-bis(3-ethylbenzothiazoline-6-sulphonic acid), and hydroxyl radical scavenging assays [134]. Additionally, PDRN exhibited antioxidant activity in lipopolysaccharide-stimulated RAW264.7 cells [135]. While the exact molecular mechanisms underlying these effects have not been fully elucidated, involvement of the nuclear factor erythroid 2-related factor 2 (NRF2) and heme oxygenase-1 (HO-1) pathways has been suggested [93,136]. Although these data stem primarily from macrophage models, which may limit comprehensive mechanistic insights specific to skin cells, RAW264.7 cells are a widely accepted in vitro model in dermatological research [137,138,139]. Moreover, the NRF2/HO-1 signaling axis is well recognized as a critical mediator of cutaneous oxidative stress responses [140,141,142]. Taken together, despite some limitations, the current body of evidence indicates that PDRN may possess antioxidant activity relevant for mitigating oxidative damage in skin-related conditions. Further studies are warranted to elucidate the precise molecular mechanisms underlying the antioxidant effects of PDRN on skin cells and to optimize its therapeutic applications for oxidative stress-related dermatological disorders.

### 3.4. Comparison of PN and PDRN

Although both PN and PDRN are classified as nucleic acid-based biopolymers, ac-cumulating evidence suggests that they serve distinct roles in dermatological applications (Table 1). PNs are generally associated with higher molecular weight and greater viscosity relative to PDRN, which is primarily recognized for its pharmacological activity, acting through defined molecular pathways such as anti-inflammatory modulation and tissue regeneration. To date, no standardized criteria have been established to delineate molecular weight thresholds that clearly distinguish PN from PDRN, despite considerable variation among previously proposed classifications [11,20,77,143]. Furthermore, these distinctions are often defined solely by manufacturers, based on proprietary technologies and production methods. The term of PN is also used inconsistently in both scientific literature and commercial labeling, frequently encompassing PDRN without clear differentiation. In some cases, formulations initially labeled as PN have subsequently been found to contain, or consist predominantly of, PDRN upon further analysis.

Despite the well-established clinical efficacy and favorable safety profiles of both PN and PDRN [144], the lack of standardized definitions and mechanistic clarity continues to obscure comparative interpretation. This highlights a critical knowledge gap and under-scores the need for rigorous, mechanistically driven research to clarify the distinct pharmacodynamic and biological properties of each compound. Based on current evidence, PN may function primarily as a structural and rheological support agent, whereas PDRN is more consistently characterized as a pharmacologically active molecule with well-defined mechanisms of action.

## 4. Clinical Uses of PN and PDRN

### 4.1. Safety of PN and PDRN

Nucleic acid–based biomolecules such as PDRN and PN have been extensively utilized in clinical settings, owing to their favorable safety and biocompatibility profiles [15,16,145,146]. PDRN has demonstrated a strong safety profile in both acute and chronic toxicity studies, where no significant immunogenic responses were observed [11,17,19]. This may be attributable to the high-temperature purification process applied to the DNA during its production [147]. To date, PDRN injections have not been clinically associated with serious adverse effects or persistent skin abnormalities [148]. Similarly, PN has also exhibited excellent tolerability in both in vitro and in vivo models, with no notable side effects reported [149]. In clinical practice, PN is widely recognized for its high biocompatibility and biodegradability and is commonly used as a skin booster component [150]. Furthermore, post-marketing surveillance and pharmacovigilance data have not revealed any significant safety concerns for either compound [19,151].

Related to this, the scope of clinical application of these compounds might vary globally because of differences in regional regulatory frameworks and approval statuses. In Korea, PDRN is approved for the treatment of graft-induced wounds and tissue regeneration [48,152,153], whereas PN is designated as a medical device [154]. In particular, the use of PN extends beyond Korea to the European Union [155] as well as other countries such as Malaysia [156], Thailand [157], Australia [158], and so on. Consequently, both PDRN and PN are extensively used by dermatologists and esthetic practitioners for skin rejuvenation and other dermatological indications.

### 4.2. Isolation and Purification of PN and PDRN for Clinical Uses

The clinical application of bioactive nucleic acids necessitates stringent isolation and purification processes to ensure high purity, sterility, and product consistency [20,159]. These polynucleotide-based agents are typically refined through proprietary multi-step protocols, often incorporating heat treatment to achieve high DNA content while minimizing residual impurities such as amino acids, low-molecular-weight peptides, and glycosaminoglycans [160]. Two primary extraction methods, namely chemical and non-organic approaches, are used for isolating PDRN from salmon sources. While the chemical method is cost-effective and technically accessible, it involves multiple steps including thawing, cell lysis, sterilization, molecular weight reduction, precipitation, and granulation, and often yields a broad molecular weight distribution [159,160,161]. In contrast, non-organic techniques use milder conditions, such as neutral lysis buffers and shorter reaction times, and thereby show improved extraction efficiency and product purity [162]. Alternative approaches using biological sources such as plants, algae, and sea cucumbers have also been explored for PDRN production, although these approaches tend to be more complex, and the products are often safeguarded by patents [134,163,164]. A notable example is Placentex^®^, a PDRN-based therapeutic developed by Mastelli S.R.L. in Italy. This product is manufactured using a proprietary High Purification Technology (HPT) designed to enhance purity and partially control the polymer length [165,166].Although HPT has improved batch consistency, fine control of product molecular weight is challenging, which often results in heterogeneity among DNA fragments [167]. The commercialization of Placentex^®^ in the early 1990s marked a significant milestone in the therapeutic use of PDRN and catalyzed advancements in DNA purification technologies [147,148,168].

More recently, HPT and similar innovations have also been adapted for PN-based products. For instance, DNA fragment Optimizing Technology (DOT™), developed by PharmaResearch Co. Ltd., has been used in the production of both PN and PDRN formulations [24,169,170,171]. Consequently, several pharmaceutical companies have established proprietary platforms for the extraction, isolation, and purification of these polynucleotides, with an emphasis on improved yield, purity, and reproducibility. These technologies are not only essential for quality assurance but have also been leveraged for branding and marketing purposes.

### 4.3. Current Status of Clinical Applications of PN

PNs have gained significant recognition for their utility in medical applications, where they are widely used as skin boosters and biopolymeric agents for anti-aging and tissue revitalization. In South Korea, PN-based injectable products are regulated as medical devices [153,154,172]. Intradermal administration of PN-based formulations enhances dermal characteristics by improving skin elasticity and hydration [157]. PNs are often combined with other biocompatible polymers, such as HA, poly-L-lactic acid, or various bioactive compounds, to achieve synergistic effects on skin texture and rejuvenation outcomes [21,24,31,32,33,34,35,40,41,42,150]. Their high biocompatibility and biodegradability, accompanied by favorable safety profiles and low immunogenicity, also make them suitable as facial fillers [22,24,31,152,161,167]. Clinically, PNs have been used to treat various facial areas, including marionette lines and the lips, providing long-lasting effects with a reduced risk of adverse events. Notably, in a randomized, double-blind, split-face trial, PN-based fillers demonstrated superior outcomes to those of HA fillers in terms of improving skin roughness, pore size, and hydration. These benefits were particularly evident in the periorbital region, where PN injections were well-tolerated and associated with minimal side effects [14,170].

The use of a PN-based medical device incorporating HPT^®^, developed by Mastelli S.R.L., significantly improved facial wrinkles and skin roughness, as measured using three-dimensional skin imaging and the global esthetic improvement scale (GAIS) [165,166]. Additional improvements were observed in skin surface evenness, including reduction in pores, wrinkles, and acne scars [168,169]. Rejuran^®^, a commercially available purified PN, has shown efficacy in treating crow’s feet, with improvements substantiated by objective measurements such as the crow’s feet grading scale (CFGS), GAIS, standardized photography, and 3D image analysis [170]. Despite the limited sample size in some reported studies, PN treatment has also yielded improvements in facial fine lines, overall skin texture, wrinkles, and tone [15,153,171,172]. A combination of PN with non-insulated microneedle radio frequency has demonstrated enhanced skin elasticity and wrinkle reduction [171]. Collectively, these findings underscore the potential usefulness of PNs in managing key clinical signs of skin aging and highlight their multifaceted utility in both esthetic and dermatologic applications.

### 4.4. Current Status of Clinical Use of PDRN

PDRNs have been approved by the Ministry of Food and Drug Safety in South Korea for use as pharmaceutical agents for tissue regeneration and wound healing, particularly for skin graft-associated injuries [19,152,153]. Consequently, they have since gained widespread clinical adoption. PDRNs are classified as prescription medications approved for injectable use for wound healing/tissue repair and knee osteoarthritis [19,173,174]. Furthermore, PDRNs are also increasingly being incorporated into cosmetic products for topical use and are registered as sodium DNA or hydrolyzed DNA [135,175]. These multifaceted applications reflect the regulatory flexibility and broad clinical utility of PDRNs across both therapeutic and esthetic domains.

PDRN-based injectable pharmaceuticals are frequently used in dermatological practice because of their multifactorial benefits, including tissue regeneration, dermal rejuvenation, hypopigmentation, wound healing, anti-inflammatory effects, anti-aging properties, and promotion of hair regrowth [23,147]. Clinical dosing regimens vary depending on the indication, and low-dose/low-level protocols are often used for cosmetic and esthetic purposes, where high-dose/high-level protocols are reserved for regenerative therapies and wound repair [19,144,176,177]. This variability required the use of individualized dosing strategies to optimize therapeutic efficacy in different clinical contexts. Multiple PDRN-based injectable products have received regulatory approval in South Korea and are actively used in dermatological clinics. These include Placentex Inj., Rejuvenex Inj., HiDr Injection, HiDr Prefilled Injection, Newdien Injection, Newcleo Injection, Poly-DN Inj., and Recovery Inj., all of which are listed by the Korea Pharmaceutical Information Center [178]. These products are widely prescribed and administered for various skin health indications, highlighting the established role of PDRN in both medical dermatology and esthetic skin care.

## 5. Discussion

Nucleic acid polymers have been widely used to enhance skin health and have attracted increasing attention in research and are being incorporated into pharmaceuticals, medical devices, and cosmetic products, contingent upon varying regional regulatory classifications. As previously noted, the same active compounds may be classified differently across countries. The scope of their applications is expanding, particularly in Italy, South Korea, and other Asian countries. Notably, numerous studies have used the terms PN and PDRN interchangeably, despite substantial differences in their molecular weight distributions and origins. This compositional heterogeneity complicates direct comparisons across studies and underscores an urgent need for standardized definitions and characterization criteria within both research and clinical contexts. For instance, proprietary technologies such as Mastelli’s HPT™ and PharmaResearch’s DOT™ have been introduced, yet detailed methodologies remain largely undisclosed. Although PN and PDRN have been extensively investigated, few studies explored alternative sources, such as plant-, microbe-, and seaweed-derived nucleotides, underscoring a substantial gap in comprehensive characterization [11,134,135,179,180,181]. There is a broad consensus on the need to develop PDRN and PN from alternative sources to overcome limitations related to seasonal availability, high production costs, and environmental sustainability. However, these efforts are in their early stages. To date, salmon- and trout-derived PDRN and PN continue to dominate dermatological applications, while research into alternative sources is ongoing.

The current review focuses primarily on the role and implications of the use of PDRN and PN in skin health management and provides a comparative analysis of extant literature. Nonetheless, several limitations were encountered in conducting this review. These included the interchangeable use of PN and PDRN in the literature, their broad molecular-weight ranges and distributions, and the varying purification methods. Furthermore, current research on PDRN and PN is largely concentrated in a limited number of countries, where regulatory frameworks and approval pathways differ. These disparities hinder cross-national comparisons and complicate the generalization of clinical outcomes.

Despite these challenges, the expanding global market and growing clinical interest in nucleic acid-based biomolecules strongly suggest that research into PDRN and PN will continue to accelerate. As ongoing and future studies produce more robust, standardized data, distinctions among the cosmetic, cosmeceutical, esthetic, and therapeutic applications are expected to become better and well-defined, enabling more targeted and evidence-based applications.

## 6. Conclusions and Perspectives

Accumulating biological and clinical data indicates that both PDRN and PN can improve the condition of the skin and promote its regeneration, thereby mitigating visible signs of skin aging. Despite differences in their molecular mechanisms and biological effects, both compounds hold substantial potential for extensive future research and development for dermatological applications. Their effectiveness is highly dependent on formulation quality and appropriate clinical use, which underscores the importance of physician oversight tailored to individual patient needs.

This review is intended to provide a resource for researchers as well as practical guidance for clinicians working on dermatological uses of PN or PDRN. As research progresses and standardized methods are established, a clearer understanding of the mechanisms, clinical benefits, and wider dermatological applications of both compounds is expected, which would contribute to supporting the development of more targeted and personalized skin therapies.

## Figures and Tables

**Table 1:** Comparative summary of the molecular characteristics, mechanisms of action, clinical uses, and regulatory status of PN and PDRN in skin health applications.

Feature	PN	PDRN
**Structural Characteristics**	Longer chain of DNA fragments; relatively higher molecular weight, clinical use of relatively lower-gauge needle (e.g., generally, 27 to 30-gauge) ^1^	Shorter chain of DNA fragments; relatively lower molecular weight, clinical use of relatively higher-gauge needle ^2^
**Water Retention/Viscoelasticity**	High water content and viscoelasticity, hydrogel formation	Lower viscoelasticity, more fluidic property
**Main Mechanism of Action**	Hydration, structural support	A2A adenosine receptor activation, nucleotide salvage pathway
**Clinical Applications**	Skin boosters, intravenous-articular injection, etc.	Wound healing, skin regeneration, anti-inflammatory, hypopigmentation, hair regrowth, etc.
**Regulatory Classification (in South Korea)**	Medical device (e.g., Rejuran^®^)	Pharmaceutical agent (e.g., Placentex^®^, Newdien Injection, PDRN Injection)

^1^ In certain formulations, the concentration is intentionally reduced to enable administration via higher-gauge needles (e.g., 32 to 34-gauge without compromising injectability); ^2^ Needle size is not strictly regulated due to the low viscosity of the formulation.

## References

[1] Walker M. (2022). Human skin through the ages. Int. J. Pharm..

[2] Menon G., Kligman A. (2009). Barrier Functions of Human Skin: A Holistic View. Ski. Pharmacol. Physiol..

[3] Arda O., Göksügür N., Tüzün Y. (2014). Basic histological structure and functions of facial skin. Clin. Dermatol..

[4] Jiao Q., Zhi L., You B., Wang G., Wu N., Jia Y. (2024). Skin homeostasis: Mechanism and influencing factors. J. Cosmet. Dermatol..

[5] Wilhelm K.P. (1991). Skin aging. Effect on transepidermal water loss, stratum corneum hydration, skin surface pH, and casual sebum content. Arch. Dermatol..

[6] Tobin D.J. (2017). Introduction to skin aging. J. Tissue Viability.

[7] Zouboulis C.C., Ganceviciene R., Liakou A.I., Theodoridis A., Elewa R., Makrantonaki E. (2019). Aesthetic aspects of skin aging, prevention, and local treatment. Clin. Dermatol..

[8] Ganceviciene R., Liakou A.I., Theodoridis A., Makrantonaki E., Zouboulis C.C. (2012). Skin anti-aging strategies. Derm.-Endocrinol..

[9] Ak M. (2019). Skin Aging & Modern Age Anti-Aging Strategies. Int. J. Clin. Dermatol. Res..

[10] Oh S., Kim Y.H., Kim B.R., Seo H.-M., Kwon S.-H., Choi H., Lee H., Na J.-I., Choi C.P., Ko J.Y. (2025). Real-World Clinical Practice on Skin Rejuvenation Among Korean Board-Certified Dermatologists: Survey-Based Results. Ann. Dermatol..

[11] Marques C., Porcello A., Cerrano M., Hadjab F., Chemali M., Lourenço K., Hadjab B., Raffoul W., Applegate L.A., Laurent A.E. (2025). From Polydeoxyribonucleotides (PDRNs) to Polynucleotides (PNs): Bridging the Gap Between Scientific Definitions, Molecular Insights, and Clinical Applications of Multifunctional Biomolecules. Biomolecules.

[12] Kim M.J., Wan J., Oksana L., Yuliia L., Chugay O., Platonova O., Sydorchuk O., Yi K.-H. (2024). Polynucleotide-based treatments for various facial scars including combat injuries. J. Dermatol. Treat..

[13] Polynucleotides Injectables Market Size-By Application, By End Use, Forecast, 2025–2034. https://www.gminsights.com/industry-analysis/polynucleotides-injectables-market.

[14] Rho N.K., Han K.H., Cho M., Kim H.S. (2023). A survey on the cosmetic use of injectable polynucleotide: The pattern of practice among Korean Dermatologists. J. Cosmet. Dermatol..

[15] Yi K., Winayanuwattikun W., Kim S., Wan J., Vachatimanont V., Putri A.I., Hidajat I.J., Yogya Y., Pamela R. (2024). Skin boosters: Definitions and varied classifications. Ski. Res. Technol..

[16] Webb W.R., Rahman E., Rao P., Abu-Farsakh H.N., Yu N., Garcia P.E., Ioannidis S., Sayed K., Tam E., Philipp-Dormston W.G. (2024). Points to ponder on the role of polynucleotides in regenerative and aesthetic medicine: A systematic review. Eur. J. Plast. Surg..

[17] Khan A., Wang G., Zhou F., Gong L., Zhang J., Qi L., Cui H. (2022). Polydeoxyribonucleotide: A promising skin anti-aging agent. Chin. J. Plast. Reconstr. Surg..

[18] Samizadeh S. (2023). Polynucleotides: The crucial biomolecules bridging therapeutics and aesthetics. J. Aesthetic Nurs..

[19] Squadrito F., Bitto A., Irrera N., Pizzino G., Pallio G., Minutoli L., Altavilla D. (2017). Pharmacological Activity and Clinical Use of PDRN. Front. Pharmacol..

[20] Shin S.M., Baek E.J., Kim K.J., Park E.J. (2023). Polydeoxyribonucleotide exerts opposing effects on ERK activity in human skin keratinocytes and fibroblasts. Mol. Med. Rep..

[21] Abuyousif H.S., Porcello A., Cerrano M., Marques C., Scaletta C., Lourenço K., Abdel-Sayed P., Chemali M., Raffoul W., Hirt-Burri N. (2025). In Vitro Evaluation and Clinical Effects of a Regenerative Complex with Non-Cross-Linked Hyaluronic Acid and a High-Molecular-Weight Polynucleotide for Periorbital Treatment. Polymers.

[22] Park K.Y., Seok J., Rho N.K., Kim B.J., Kim M.N. (2015). Long-chain polynucleotide filler for skin rejuvenation: Efficacy and complications in five patients. Dermatol. Ther..

[23] Galeano M., Pallio G., Irrera N., Mannino F., Bitto A., Altavilla D., Vaccaro M., Squadrito G., Arcoraci V., Colonna M.R. (2021). Polydeoxyribonucleotide: A Promising Biological Platform to Accelerate Impaired Skin Wound Healing. Pharmaceuticals.

[24] Kim M.J., Park H., Jung R., Won C., Ohk S., Kim H., Roh N., Yi K. (2024). High-resolution 3-D scanning electron microscopy (SEM) images of DOT™ polynucleotides (PN): Unique scaffold characteristics and potential applications in biomedicine. Ski. Res. Technol..

[25] Kim M.J., Park H., Oh S.M., Yi K. (2023). Polynucleotide injection treatment for iatrogenic fat atrophy in two patients: Potential for safe volumization in aesthetic medicine. Ski. Res. Technol..

[26] Colangelo M.T., Belletti S., Govoni P., Guizzardi S., Galli C. (2021). A Biomimetic Polynucleotides–Hyaluronic Acid Hydrogel Promotes Wound Healing in a Primary Gingival Fibroblast Model. Appl. Sci..

[27] Pitassi L.H.U., Pearson K., de Assis L.A., Biesman B., Calomeni M., Bay-Aguilera S.D., Wyles S.P. (2024). Polynucleotides in Skin Regeneration: Targeting the Adenosine A2A Receptor and Salvage Pathway. Dermatol. Surg..

[28] Cavallini M., Bartoletti E., Maioli L., Palmieri I.P., Papagni M., Priori M., Trocchi G. (2024). Value and Benefits of the Polynucleotides HPT™ Dermal Priming Paradigm A Consensus on Practice Guidance for Aesthetic Medicine Practitioners and Future Research. Clin. Exp. Dermatol. Ther..

[29] Lee D., Choi H., Yoo K., Park Y.J., Park H.J., Oh S.M., Ji G.H., Rah G.C., Shin D.W. (2024). Assessment of current practices and perceived effectiveness of injectable polynucleotide for enlarged facial pores among cosmetic physicians: A survey-based evaluation. Ski. Res. Technol..

[30] Oh N., Hwang J., Kang M.S., Yoo C.-Y., Kwak M., Han D.-W. (2025). Versatile and Marvelous Potentials of Polydeoxyribonucleotide for Tissue Engineering and Regeneration. Biomater. Res..

[31] Kim H.T., Lee Y.J., Paik S.H., Moon Y.S., Lee W.J., Chang S.E., Lee M.W., Choi J.H., Jung J.M., Won C.H. (2020). Comparison of the effects of polynucleotide and hyaluronic acid fillers on periocular rejuvenation: A randomized, double-blind, split-face trial. J. Dermatol. Treat..

[32] Ha Y.J., Tak K.H., Jung J., Lee J.L., Kim C.W., Ah Y., Kim S., Moon I.J., Yoon Y.S. (2024). The Effect of Polynucleotide-Hyaluronic Acid Hydrogel in the Recovery After Mechanical Skin Barrier Disruption. Ski. Res. Technol..

[33] Titcomb L. (2025). A literature review on polynucleotide efficacy on skin rejuvenation, and review of the regulatory status and guidelines around polynucleotides. J. Aesthetic Nurs..

[34] Choi S.H., Kim H.C., Jang S.G., Lee Y.J., Heo J.Y., Kweon G.R., Ryu M.J. (2024). Effects of a Combination of Polynucleotide and Hyaluronic Acid for Treating Osteoarthritis. Int. J. Mol. Sci..

[35] Heo T.-H., Gu B.K., Ohk K., Yoon J.-K., Son Y.H., Chun H.J., Yang D.-H., Jeong G.-J. (2025). Polynucleotide and Hyaluronic Acid Mixture for Skin Wound Dressing for Accelerated Wound Healing. Tissue Eng. Regen. Med..

[36] Dallari D., Sabbioni G., Del Piccolo N., Carubbi C., Veronesi F., Torricelli P., Fini M. (2020). Efficacy of Intra-Articular Polynucleotides Associated With Hyaluronic Acid Versus Hyaluronic Acid Alone in the Treatment of Knee Osteoarthritis: A Randomized, Double-Blind, Controlled Clinical Trial. Am. J. Ther..

[37] Vanelli R., Costa P., Rossi S.M.P., Benazzo F. (2010). Efficacy of intra-articular polynucleotides in the treatment of knee osteoarthritis: A randomized, double-blind clinical trial. Knee Surg. Sports Traumatol. Arthrosc..

[38] Segreto F., Carotti S., Marangi G.F., Francesconi M., Scaramuzzino L., Gratteri M., Caldaria E., Morini S., Persichetti P. (2020). The use of acellular porcine dermis, hyaluronic acid and polynucleotides in the treatment of cutaneous ulcers: Single blind randomised clinical trial. Int. Wound J..

[39] Stagni C., Rocchi M., Mazzotta A., Del Piccolo N., Rani N., Govoni M., Vivarelli L., Veronesi F., Fini M., Dallari D. (2021). Randomised, double-blind comparison of a fixed co-formulation of intra-articular polynucleotides and hyaluronic acid versus hyaluronic acid alone in the treatment of knee osteoarthritis: Two-year follow-up. BMC Musculoskelet. Disord..

[40] Kim J.H., Kwon T.-R., Lee S.E., Na Jang Y., Han H.S., Mun S.K., Kim B.J. (2020). Comparative Evaluation of the Effectiveness of Novel Hyaluronic Acid-Polynucleotide Complex Dermal Filler. Sci. Rep..

[41] Draelos Z.D. (2012). New treatments for restoring impaired epidermal barrier permeability: Skin barrier repair creams. Clin. Dermatol..

[42] Migliore A. (2015). Effectiveness and utility of hyaluronic acid in osteoarthritis. Bone Abstr..

[43] Kuppa S.S., Kim H.-K., Kang J.-Y., Lee S.-C., Yang H.-Y., Sankaranarayanan J., Seon J.-K. (2023). Polynucleotides Suppress Inflammation and Stimulate Matrix Synthesis in an In Vitro Cell-Based Osteoarthritis Model. Int. J. Mol. Sci..

[44] Kim T.W., Chang M.J., Shin C.Y., Chang C.B., Kang S.-B. (2023). A randomized controlled trial for comparing efficacy and safety between intraarticular polynucleotide and hyaluronic acid for knee osteoarthritis treatment. Sci. Rep..

[45] Bowman S., Awad M.E., Hamrick M.W., Hunter M., Fulzele S. (2018). Recent advances in hyaluronic acid based therapy for osteoarthritis. Clin. Transl. Med..

[46] Moon J.Y., Kim J., Lee J.Y., Ko Y., Park H.J., Jeon Y.H. (2022). Comparison of Polynucleotide, Sodium Hyaluronate, and Crosslinked Sodium Hyaluronate for the Management of Painful Knee Osteoarthritis: A Multi-Center, Randomized, Double-Blind, Parallel-Group Study. Pain Med..

[47] Lee D., Kim W.-H., Ha J.H., Kim H., Kim J., Shin D.W. (2025). Current Practices and Perceived Effectiveness of Clinicians Regarding Polynucleotide Injection for Knee Osteoarthritis: A Survey-Based Evaluation. Healthcare.

[48] Kim T.-H., Heo S.-Y., Oh G.-W., Heo S.-J., Jung W.-K. (2021). Applications of Marine Organism-Derived Polydeoxyribonucleotide: Its Potential in Biomedical Engineering. Mar. Drugs.

[49] Kim T.-Y., Kim Y.-T., Hwang J.-T. (2024). Clinical Updates in Polydeoxyribonucleotide Injection. J. Korean Orthop. Assoc..

[50] Shin D.Y., Park J.-U., Choi M.-H., Kim S., Kim H.-E., Jeong S.-H. (2020). Polydeoxyribonucleotide-delivering therapeutic hydrogel for diabetic wound healing. Sci. Rep..

[51] Akaberi S.M., Sharma K., Ahmadi-Ashtiani H.R., Hedayati M. (2025). Polydeoxyribonucleotide in Skincare and Cosmetics: Mechanisms, Therapeutic Applications, and Advancements Beyond Wound Healing and Anti-aging. J. Ski. Stem Cell.

[52] Thellung S., Florio T., Maragliano A., Cattarini G., Schettini G. (1999). Polydeoxyribonucleotides enhance the proliferation of human skin fibroblasts: Involvement of A2 purinergic receptor subtypes. Life Sci..

[53] Guizzardi S., Galli C., Govoni P., Boratto R., Cattarini G., Martini D., Belletti S., Scandroglio R. (2003). Polydeoxyribonucleotide (PDRN) promotes human osteoblast proliferation: A new proposal for bone tissue repair. Life Sci..

[54] Koo Y., Yun Y. (2016). Effects of polydeoxyribonucleotides (PDRN) on wound healing: Electric cell-substrate impedance sensing (ECIS). Mater. Sci. Eng. C.

[55] Altavilla D., Bitto A., Polito F., Marini H., Minutoli L., Stefano V., Irrera N., Cattarini G., Squadrito F. (2009). Polydeoxyribonucleotide (PDRN): A Safe Approach to Induce Therapeutic Angiogenesis in Peripheral Artery Occlusive Disease and in Diabetic Foot Ulcers. Cardiovasc. Hematol. Agents Med. Chem..

[56] Kim T., Heo S., Han J.S., Jung W. (2023). Anti-inflammatory effect of polydeoxyribonucleotides (PDRN) extracted from red alga (*Porphyra* sp.) (Ps-PDRN) in RAW 264.7 macrophages stimulated with *Escherichia coli* lipopolysaccharides: A comparative study with commercial PDRN. Cell Biochem. Funct..

[57] Sheth S., Brito R., Mukherjea D., Rybak L.P., Ramkumar V. (2014). Adenosine Receptors: Expression, Function and Regulation. Int. J. Mol. Sci..

[58] Sebastiã£O A. (2000). Fine-tuning neuromodulation by adenosine. Trends Pharmacol. Sci..

[59] Cunha R. (2001). Adenosine as a neuromodulator and as a homeostatic regulator in the nervous system: Different roles, different sources and different receptors. Neurochem. Int..

[60] de Mendonça A., Ribeiro J.A. (2001). Adenosine and synaptic plasticity. Drug Dev. Res..

[61] Li J.-M., Fenton R.A., Wheeler H., Powell C.C., Peyton B.D., Cutler B.S., Dobson J.G. (1998). Adenosine A2aReceptors Increase Arterial Endothelial Cell Nitric Oxide. J. Surg. Res..

[62] Haskó G., Pacher P. (2007). A2A receptors in inflammation and injury: Lessons learned from transgenic animals. J. Leukoc. Biol..

[63] Odashima M., Otaka M., Jin M., Komatsu K., Wada I., Matsuhashi T., Horikawa Y., Hatakeyama N., Oyake J., Ohba R. (2005). Selective adenosine A_2A_ receptor agonist, ATL-146e, attenuates stress-induced gastric lesions in rats. J. Gastroenterol. Hepatol..

[64] Biaggioni I., Paul S., Puckett A., Arzubiaga C. (1991). Caffeine and theophylline as adenosine receptor antagonists in humans. J. Pharmacol. Exp. Ther..

[65] Saini A., Patel R., Gaba S., Singh G., Gupta G., Monga V. (2022). Adenosine receptor antagonists: Recent advances and therapeutic perspective. Eur. J. Med. Chem..

[66] Baek A., Kim M., Kim S.H., Cho S.-R., Kim H.J. (2018). Anti-inflammatory Effect of DNA Polymeric Molecules in a Cell Model of Osteoarthritis. Inflammation.

[67] Vincenzi F., Pasquini S., Contri C., Cappello M., Nigro M., Travagli A., Merighi S., Gessi S., Borea P.A., Varani K. (2023). Pharmacology of Adenosine Receptors: Recent Advancements. Biomolecules.

[68] Jacobson K.A., Gao Z.-G. (2006). Adenosine receptors as therapeutic targets. Nat. Rev. Drug Discov..

[69] Säve S., Persson K. (2010). Effects of Adenosine A2A and A2B Receptor Activation on Signaling Pathways and Cytokine Production in Human Uroepithelial Cells. Pharmacology.

[70] Bitto A., Polito F., Irrera N., D’AScola A., Avenoso A., Nastasi G., Campo G.M., Micali A., Bagnato G., Minutoli L. (2011). Polydeoxyribonucleotide reduces cytokine production and the severity of collagen-induced arthritis by stimulation of adenosine A_2A_ receptor. Arthritis Rheum..

[71] Colangelo M.T., Galli C., Guizzardi S. (2020). The Effects of Polydeoxyribonucleotide on Wound Healing and Tissue Regeneration: A Systematic Review of the Literature. Regen. Med..

[72] Kim D.-S., Lee J.-K., Jung J.-W., Baek S.-W., Kim J.H., Heo Y., Kim T.-H., Han D.K. (2021). Promotion of Bone Regeneration Using Bioinspired PLGA/MH/ECM Scaffold Combined with Bioactive PDRN. Materials.

[73] Cámara Y., González-Vioque E., Scarpelli M., Torres-Torronteras J., Martí R. (2013). Feeding the deoxyribonucleoside salvage pathway to rescue mitochondrial DNA. Drug Discov. Today.

[74] Nyhan W.L. (2001). Nucleotide Synthesis via Salvage Pathway. Encyclopedia of Life Sciences (eLS).

[75] Jeong W., Yang C.E., Roh T.S., Kim J.H., Lee J.H., Lee W.J. (2017). Scar Prevention and Enhanced Wound Healing Induced by Polydeoxyribonucleotide in a Rat Incisional Wound-Healing Model. Int. J. Mol. Sci..

[76] Bitto A., Galeano M., Squadrito F., Minutoli L., Polito F., Dye J.F., Clayton E.A.F., Calò M., Venuti F.S., Vaccaro M. (2008). Polydeoxyribonucleotide improves angiogenesis and wound healing in experimental thermal injury. Crit. Care Med..

[77] Hwang K., Kim J., Park E.Y., Cha S. (2018). An effective range of polydeoxyribonucleotides is critical for wound healing quality. Mol. Med. Rep..

[78] Bristow R.G., Hill R.P. (2008). Hypoxia, DNA repair and genetic instability. Nat. Rev. Cancer.

[79] Tran D.H., Kim D., Kesavan R., Brown H., Dey T., Soflaee M.H., Vu H.S., Tasdogan A., Guo J., Bezwada D. (2024). De novo and salvage purine synthesis pathways across tissues and tumors. Cell.

[80] D’Andrea F., Mosella F., Maruccia M., Papa G., Ricci E., Giudice G. (2023). Bioinductive Dressing. Pearls and Pitfalls in Skin Ulcer Management.

[81] Wei M., He X., Liu N., Deng H. (2024). Role of reactive oxygen species in ultraviolet-induced photodamage of the skin. Cell Div..

[82] D’eRrico M., Lemma T., Calcagnile A., De Santis L.P., Dogliotti E. (2007). Cell type and DNA damage specific response of human skin cells to environmental agents. Mutat. Res. Mol. Mech. Mutagen..

[83] Schumacher B., Pothof J., Vijg J., Hoeijmakers J.H.J. (2021). The central role of DNA damage in the ageing process. Nature.

[84] Yousefzadeh M., Henpita C., Vyas R., Soto-Palma C., Robbins P., Niedernhofer L. (2021). DNA damage—How and why we age?. eLife.

[85] Farage M.A., Miller K.W., Elsner P., Maibach H.I. (2008). Intrinsic and extrinsic factors in skin ageing: A review. Int. J. Cosmet. Sci..

[86] El-Domyati M., Attia S., Saleh F., Brown D., Birk D.E., Gasparro F., Ahmad H., Uitto J. (2002). Intrinsic aging vs. photoaging: A comparative histopathological, immunohistochemical, and ultrastructural study of skin. Exp. Dermatol..

[87] Krutmann J., Schikowski T., Morita A., Berneburg M. (2021). Environmentally-Induced (Extrinsic) Skin Aging: Exposomal Factors and Underlying Mechanisms. J. Investig. Dermatol..

[88] Yu M., Lee J.Y. (2016). Polydeoxyribonucleotide improves wound healing of fractional laser resurfacing in rat model. J. Cosmet. Laser Ther..

[89] Tharaux P.-L., Chatziantoniou C., Fakhouri F., Dussaule J.-C. (2000). Angiotensin II Activates Collagen I Gene Through a Mechanism Involving the MAP/ER Kinase Pathway. Hypertension.

[90] Belletti S., Uggeri J., Gatti R., Govoni P., Guizzardi S. (2007). Polydeoxyribonucleotide promotes cyclobutane pyrimidine dimer repair in UVB-exposed dermal fibroblasts. Photodermatol. Photoimmunol. Photomed..

[91] Ishii T., Asuwa N. (2000). Collagen and elastin degradation by matrix metalloproteinases and tissue inhibitors of matrix metalloproteinase in aortic dissection. Hum. Pathol..

[92] Labat-Robert J., Fourtanier A., Boyer-Lafargue B., Robert L. (2000). Age dependent increase of elastase type protease activity in mouse skin. J. Photochem. Photobiol. B Biol..

[93] Kim H.M., Byun K.-A., Oh S., Yang J.Y., Park H.J., Chung M.S., Son K.H., Byun K. (2022). A Mixture of Topical Forms of Polydeoxyribonucleotide, Vitamin C, and Niacinamide Attenuated Skin Pigmentation and Increased Skin Elasticity by Modulating Nuclear Factor Erythroid 2-like 2. Molecules.

[94] Park J.M., Nam G.B., Lee E.-S., Kim H.-M., Kim H., Myoung K., Lee J.E., Baek H.S., Ko J., Lee C.S. (2025). Effects of Chlorella protothecoides-derived polydeoxyribonucleotides on skin regeneration and wound healing. Arch. Dermatol. Res..

[95] Lin J.Y., Fisher D.E. (2007). Melanocyte biology and skin pigmentation. Nature.

[96] Moreiras H., Seabra M.C., Barral D.C. (2021). Melanin Transfer in the Epidermis: The Pursuit of Skin Pigmentation Control Mechanisms. Int. J. Mol. Sci..

[97] Lu Y., Tonissen K.F., Di Trapani G. (2021). Modulating skin colour: Role of the thioredoxin and glutathione systems in regulating melanogenesis. Biosci. Rep..

[98] Kim J.C., Park T.J., Kang H.Y. (2022). Skin-Aging Pigmentation: Who Is the Real Enemy?. Cells.

[99] Yoo J. (2021). Differential diagnosis and management of hyperpigmentation. Clin. Exp. Dermatol..

[100] Ladizinski B., Mistry N., Kundu R.V. (2011). Widespread Use of Toxic Skin Lightening Compounds: Medical and Psychosocial Aspects. Dermatol. Clin..

[101] Noh T.K., Chung B.Y., Kim S.Y., Lee M.H., Kim M.J., Youn C.S., Lee M.W., Chang S.E. (2016). Novel Anti-Melanogenesis Properties of Polydeoxyribonucleotide, a Popular Wound Healing Booster. Int. J. Mol. Sci..

[102] Song L., Zhang S. (2023). Anti-Aging Activity and Modes of Action of Compounds from Natural Food Sources. Biomolecules.

[103] Kim Y.-J., Kim M.-J., Kweon D.-K., Lim S.-T., Lee S.-J. (2019). Polydeoxyribonucleotide Activates Mitochondrial Biogenesis but Reduces MMP-1 Activity and Melanin Biosynthesis in Cultured Skin Cells. Appl. Biochem. Biotechnol..

[104] Lima T.d.P.d.L., Passos M.F. (2021). Skin wounds, the healing process, and hydrogel-based wound dressings: A short review. J. Biomater. Sci. Polym. Ed..

[105] Kirsner R.S., Eaglstein W.H. (1993). The Wound Healing Process. Dermatol. Clin..

[106] Peña O.A., Martin P. (2024). Cellular and molecular mechanisms of skin wound healing. Nat. Rev. Mol. Cell Biol..

[107] Dryden M.S. (2010). Complicated skin and soft tissue infection. J. Antimicrob. Chemother..

[108] Lin X., Lai Y. (2024). Scarring Skin: Mechanisms and Therapies. Int. J. Mol. Sci..

[109] Choudhary V., Choudhary M., Bollag W.B. (2024). Exploring Skin Wound Healing Models and the Impact of Natural Lipids on the Healing Process. Int. J. Mol. Sci..

[110] Wells A., Nuschke A., Yates C.C. (2016). Skin tissue repair: Matrix microenvironmental influences. Matrix Biol..

[111] Varano F., Catarzi D., Vigiani E., Calenda S., Colotta V. (2023). Adenosine Receptor Ligands as Potential Therapeutic Agents for Impaired Wound Healing and Fibrosis. Purinergic Receptors and Their Modulators.

[112] Kwon T.-R., Han S.W., Kim J.H., Lee B.C., Kim J.M., Hong J.Y., Kim B.J. (2019). Polydeoxyribonucleotides Improve Diabetic Wound Healing in Mouse Animal Model for Experimental Validation. Ann. Dermatol..

[113] Baek A., Kim Y., Lee J.W., Lee S.C., Cho S.-R. (2018). Effect of Polydeoxyribonucleotide on Angiogenesis and Wound Healing in an In Vitro Model of Osteoarthritis. Cell Transplant..

[114] Veronesi F., Dallari D., Sabbioni G., Carubbi C., Martini L., Fini M. (2017). Polydeoxyribonucleotides (PDRNs) From Skin to Musculoskeletal Tissue Regeneration via Adenosine A_2A_ Receptor Involvement. J. Cell. Physiol..

[115] Lai-Cheong J.E., McGrath J.A. (2013). Structure and function of skin, hair and nails. Medicine.

[116] Romanovsky A.A. (2014). Skin temperature: Its role in thermoregulation. Acta Physiol..

[117] Lunenfeld B., Gooren L.J.G., Morales A. (2008). Hormone treatment and preventative strategies in aging men: Whom to treat, when to treat and how to treat. Textbook of Men’s Health and Aging.

[118] Alessandrini A., Bruni F., Piraccini B., Starace M. (2020). Common causes of hair loss–clinical manifestations, trichoscopy and therapy. J. Eur. Acad. Dermatol. Venereol..

[119] Liu D., Xu Q., Meng X., Liu X., Liu J. (2024). Status of research on the development and regeneration of hair follicles. Int. J. Med. Sci..

[120] Lee S., Zheng Z., Kang J., Kim D., Oh S.H., Bin Cho S. (2015). Therapeutic efficacy of autologous platelet-rich plasma and polydeoxyribonucleotide on female pattern hair loss. Wound Repair Regen..

[121] Bin Cho S., Zheng Z., Kang J.-S., Kim H. (2016). Therapeutic Efficacy of 1,927-nm Fractionated Thulium Laser Energy and Polydeoxyribonucleotide on Pattern Hair Loss. Med. Lasers.

[122] Choi Y.J., Cho S., Kim Y.K., Kim D.S. (2017). Improvement of Hair Graying during a Treatment of Male Pattern Hair Loss Using 1,927-nm Fractionated Thulium Laser Energy and Polydeoxyribonucleotide Injections. Med. Lasers.

[123] Ma L., Chen M., Fa Z., Pan W., Liao W., Gao X.-H., Huo W., Yang Y., Chen H.-D., Holahan H.M. (2017). Skin diseases caused by factors from the environment. Skin Diseases Caused by Factors from the Environment.

[124] Agrawal R., Hu A., Bollag W.B. (2023). The Skin and Inflamm-Aging. Biology.

[125] Harvanová G., Duranková S., Bernasovská J. (2023). The role of cytokines and chemokines in the inflammatory response. Alergol. Pol.-Pol. J. Allergol..

[126] Colangelo M.T., Galli C., Guizzardi S. (2020). Polydeoxyribonucleotide Regulation of Inflammation. Adv. Wound Care.

[127] Castellini C., Belletti S., Govoni P., Guizzardi S. (2017). Anti Inflammatory Property of PDRN—An in Vitro Study on Cultured Macrophages. Adv. Biosci. Biotechnol..

[128] Irrera N., Bitto A., Vaccaro M., Mannino F., Squadrito V., Pallio G., Arcoraci V., Minutoli L., Ieni A., Lentini M. (2020). PDRN, a Bioactive Natural Compound, Ameliorates Imiquimod-Induced Psoriasis through NF-κB Pathway Inhibition and Wnt/β-Catenin Signaling Modulation. Int. J. Mol. Sci..

[129] Lee H.Y., Kim D.-S., Hwang G.Y., Lee J.-K., Jung J.-W., Hwang S.Y., Baek S.-W., Yoon S.L., Ha Y., Kim K.N. (2023). Multi-modulation of immune-inflammatory response using bioactive molecule-integrated PLGA composite for spinal fusion. Mater. Today Bio.

[130] Papaccio F., D′Arino A., Caputo S., Bellei B. (2022). Focus on the Contribution of Oxidative Stress in Skin Aging. Antioxidants.

[131] Poljšak B., Dahmane R.G., Godić A. (2012). Intrinsic skin aging: The role of oxidative stress. Acta Dermatovenerol. Alp. Panon. Adriat..

[132] Poljšak B., Dahmane R. (2012). Free Radicals and Extrinsic Skin Aging. Dermatol. Res. Pr..

[133] Liu H.-M., Cheng M.-Y., Xun M.-H., Zhao Z.-W., Zhang Y., Tang W., Cheng J., Ni J., Wang W. (2023). Possible Mechanisms of Oxidative Stress-Induced Skin Cellular Senescence, Inflammation, and Cancer and the Therapeutic Potential of Plant Polyphenols. Int. J. Mol. Sci..

[134] Shu Z., Ji Y., Liu F., Jing Y., Jiao C., Li Y., Zhao Y., Wang G., Zhang J. (2024). Proteomics Analysis of the Protective Effect of Polydeoxyribonucleotide Extracted from Sea Cucumber (*Apostichopus japonicus*) Sperm in a Hydrogen Peroxide-Induced RAW264.7 Cell Injury Model. Mar. Drugs.

[135] Chae D., Oh S.-W., Choi Y.-S., Kang D.-J., Park C.-W., Lee J., Seo W.-S. (2025). First Report on Microbial-Derived Polydeoxyribonucleotide: A Sustainable and Enhanced Alternative to Salmon-Based Polydeoxyribonucleotide. Curr. Issues Mol. Biol..

[136] Pittala V., Vanella L., Salerno L., Romeo G., Marrazzo A., Di Giacomo C., Sorrenti V. (2018). Effects of Polyphenolic Derivatives on Heme Oxygenase-System in Metabolic Dysfunctions. Curr. Med. Chem..

[137] Kim J.-S., Lee E.-B., Choi J.-H., Jung J., Jeong U.-Y., Bae U.-J., Jang H.-H., Park S.-Y., Cha Y.-S., Lee S.-H. (2023). Antioxidant and Immune Stimulating Effects of *Allium cepa* Skin in the RAW 264.7 Cells and in the C57BL/6 Mouse Immunosuppressed by Cyclophosphamide. Antioxidants.

[138] Chung E., Choi H., Lim J.E., Son Y. (2014). Development of skin inflammation test model by co-culture of reconstituted 3D skin and RAW264.7 cells. Tissue Eng. Regen. Med..

[139] Lee H., Hwang D., Lee M., Lee J., Cho S., Kim T.-J., Kim H.S. (2022). Micro-Current Stimulation Suppresses Inflammatory Responses in Peptidoglycan-Treated Raw 264.7 Macrophages and *Propionibacterium acnes*-Induced Skin Inflammation via TLR2/NF-κB Signaling Pathway. Int. J. Mol. Sci..

[140] Gęgotek A., Skrzydlewska E. (2015). The role of transcription factor Nrf2 in skin cells metabolism. Arch. Dermatol. Res..

[141] Yang H.-L., Chen S.-J., Yeh J.-T., Vadivalagan C., Chiu J.-H., Hseu J.-H., Hseu Y.-C. (2024). The anti-melanogenesis, anti-photoaging, and anti-inflammation of coenzyme Q0, a major quinone derivative from Antrodia camphorata, through antioxidant Nrf2 signaling pathways in UVA/B-irradiated keratinocytes. J. Funct. Foods.

[142] Boo Y.C. (2020). Natural Nrf2 Modulators for Skin Protection. Antioxidants.

[143] Tonello G., Daglio M., Zaccarelli N., Sottofattori E., Mazzei M., Balbi A. (1996). Characterization and quantitation of the active polynucleotide fraction (PDRN) from human placenta, a tissue repair stimulating agent. J. Pharm. Biomed. Anal..

[144] Kim J.K., Chung J.Y. (2015). Effectiveness of polydeoxyribonucleotide injection versus normal saline injection for treatment of chronic plantar fasciitis: A prospective randomised clinical trial. Int. Orthop..

[145] Chai A.C., Siegwart D.J., Wang R.C. (2024). Nucleic Acid Therapy for the Skin. J. Investig. Dermatol..

[146] Araco A., Araco F. (2021). Preliminary Prospective and Randomized Study of Highly Purified Polynucleotide vs Placebo in Treatment of Moderate to Severe Acne Scars. Aesthetic Surg. J..

[147] Nguyen T.H., Wang S.-L., Nguyen V.B. (2024). Recent advances on polydeoxyribonucleotide extraction and its novel application in cosmeceuticals. Int. J. Biol. Macromol..

[148] Rubegni P., De Aloe G., Mazzatenta C., Cattarini L., Fimiani M. (2001). Clinical Evaluation of the Trophic Effect of Polydeoxyribonucleotide (PDRN) in Patients Undergoing Skin Explants. A Pilot Study. Curr. Med. Res. Opin..

[149] Lampridou S., Bassett S., Cavallini M., Christopoulos G. (2024). The Effectiveness of Polynucleotides in Esthetic Medicine: A Systematic Review. J. Cosmet. Dermatol..

[150] Oh H., Lee S., Na J., Kim J.H. (2021). Comparative Evaluation of Safety and Efficacy of a Novel Hyaluronic Acid-polynucleotide/Poly-L-lactic Acid Composite Dermal Filler. Aesthetic Plast. Surg..

[151] Borea P.A., Gessi S., Merighi S., Vincenzi F., Varani K. (2018). Pharmacology of Adenosine Receptors: The State of the Art. Physiol. Rev..

[152] Drug Approval by MFDS, Drug Information, Announcement of Drug Master File (DMF) Registration: Search by ingredient-Sodium polydeoxyribonucleotide. https://nedrug.mfds.go.kr/.

[153] EMEDI: Information Search-REJURAN. https://emedi.mfds.go.kr/search/data/MNU20237#item.

[154] EMEDI: Information Search-Polydeoxyribonucleotide. https://emedi.mfds.go.kr/search/data/MNU20237#item.

[155] Lim T.S., Liew S., Tee X.J., Chong I., Lo F.J., Ho M.J., Ong K., Cavallini M. (2024). Polynucleotides HPT for Asian Skin Regeneration and Rejuvenation. Clin. Cosmet. Investig. Dermatol..

[156] Malaysia Medical Device Register (MMDR)-Public Search: Rejuran. https://mdar.mda.gov.my/frontend/web/index.php?r=carian%2Findex&CompletedApplicationAllmdrSearch%5BglobalSearch%5D=Rejuran.

[157] Therapeutic Goods Administration (TGA)-REJURAN. https://www.tga.gov.au/resources/artg/412630.

[158] Lee D., Kim M.J., Park H.J., Rah G.C., Choi H., Anh S., Ji G.H., Kim M.S., Kim G., Shin D.W. (2023). Current practices and perceived effectiveness of polynucleotides for treatment of facial erythema by cosmetic physicians. Ski. Res. Technol..

[159] DeCollibus D.P., Searcy J., Tivesten A., Akhtar N., Lindenberg C., Abarrou N., Pradhan S., Fiandaca M., Franklin J., Govindan G. (2023). Considerations for the Terminal Sterilization of Oligonucleotide Drug Products. Nucleic Acid Ther..

[160] Yi K.-H., Park M.-S., Ree Y.-S., Kim H.M. (2023). A Review on “Skin Boosters”: Hyaluronic Acid, Poly-L-lactic Acid and Pol-D-lactic Acid, Polydeoxyribonucleotide, Polynucleotides, Growth Factor, and Exosome. Korean Assoc. Laser Dernatology Trichology.

[161] Rho N.-K., Kim H.-S., Kim S.-Y., Lee W. (2024). Injectable “Skin Boosters” in Aging Skin Rejuvenation: A Current Overview. Arch. Plast. Surg..

[162] Gupta N. (2019). DNA extraction and polymerase chain reaction. J. Cytol..

[163] Cheng L.-Y., Sun X.-M., Tang M.-Y., Jin R., Cui W.-G., Zhang Y.-G. (2016). An update review on recent skin fillers. Plast. Aesthetic Res..

[164] Cavallini M., Bartoletti E., Maioli L., Massirone A., Palmieri I.P., Papagni M., Priori M., Trocchi G., members of The Polynucleotides HPT™ Priming Board, Collegio Italiano delle Società Scientifiche di Medicina Estetica (Italian College of the Aesthetic Medicine Scientific Societies)—SIME, AGORÀ, SIES (2020). Consensus report on the use of PN-HPT™ (polynucleotides highly purified technology) in aesthetic medicine. J. Cosmet. Dermatol..

[165] Lee J.H., Han J.W., Byun J.H., Lee W.M., Kim M.H., Wu W.H. (2018). Comparison of wound healing effects between Oncorhynchus keta-derived polydeoxyribonucleotide (PDRN) and Oncorhynchus mykiss-derived PDRN. Arch. Craniofacial Surg..

[166] Palmieri I.P., Moro L., Fraone N., De Luca C., Prussia C. (2023). An Innovative PN HPT™-based Medical Device for the Therapy of Deteriorated Periocular Skin Quality. Surg. Res..

[167] Nabila I. (2024). Literature Review Mikroinjeksi Deoxyribonucleic Acid (DNA) Salmon Sebagai Agen Peremajaan Kulit Wajah. Ph.D. Thesis.

[168] Araco A., Araco F., Raichi M. (2022). Clinical efficacy and safety of polynucleotides highly purified technology (PN-HPT^®^) and cross-linked hyaluronic acid for moderate to severe nasolabial folds: A prospective, randomized, exploratory study. J. Cosmet. Dermatol..

[169] Kim J.H., Jeong J.J., Lee Y.I., Lee W.J., Lee C., Chung W.Y., Nam K., Lee J.H. (2018). Preventive effect of polynucleotide on post-thyroidectomy scars: A randomized, double-blinded, controlled trial. Lasers Surg. Med..

[170] Yogya Y., Wanitphakdeedecha R., Wongdama S., Nanchaipruek Y., Yan C., Rakchart S. (2022). Efficacy and Safety of Using Noninsulated Microneedle Radiofrequency Alone versus in Combination with Polynucleotides for Treatment of Periorbital Wrinkles. Dermatol. Ther..

[171] Hong J.Y., Lee Y.H., Kim H., Park K.Y. (2024). Therapeutic Performance of Needle Injection Versus Needle-Free Jet Injector System for Polynucleotide Filler in Skin Rejuvenation. J. Cosmet. Dermatol..

[172] Choi S.Y., Koh Y.G., Yoo K.H., Han H.S., Seok J., Kim B.J. (2024). A Randomized, Participant- and Evaluator-Blinded, Matched-Pair, Prospective Study Comparing the Safety and Efficacy Between Polycaprolactone and Polynucleotide Fillers in the Correction of Crow’s Feet. J. Cosmet. Dermatol..

[173] Kim M.S., Cho R.K., In Y. (2019). The efficacy and safety of polydeoxyribonucleotide for the treatment of knee osteoarthritis. Medicine.

[174] Jo S., Baek A., Cho Y., Kim S.H., Baek D., Hwang J., Cho S.-R., Kim H.J. (2023). Therapeutic effects of polydeoxyribonucleotide in an in vitro neuronal model of ischemia/reperfusion injury. Sci. Rep..

[175] Cosmetic Ingredient Dictionary by the Korean Cosmetic Association. https://kcia.or.kr/cid/search/ingd_view.php?no=1048.

[176] Kim B.R., Kwon S.H., Kim J.W., Jeong W.-J., Cha W., Jung Y.H., Na J.-I., Huh C.-H., Shin J.-W. (2023). Early Postoperative Injections of Polydeoxyribonucleotide Prevent Hypertrophic Scarring After Thyroidectomy: A Randomized Controlled Trial. Adv. Wound Care.

[177] Kim J.H., Kim E.S., Kim S.W., Hong S.P., Kim J. (2022). Effects of Polynucleotide Dermal Filler in the Correction of Crow’s Feet Using an Antera Three-Dimensional Camera. Aesthetic Plast. Surg..

[178] Korea Pharmaceutical Information Center (KPIC): Drug Information Search—PDRN. https://www.health.kr/.

[179] Kim D., Kim W.-J., Lee H.-K., Kwon Y.-S., Choi Y.-M. (2023). Efficacy in vitro Antioxidation and in vivo Skin Barrier Recovery of Composition Containing Mineral-cation-phyto DNA Extracted from Aloe vera Adventitious Root. Asian J. Beauty Cosmetol..

[180] Song M.H., Choi M.H., Jeong J.H., Lee S.S., Jeong W.Y. (2022). Efficiency of PDNR (Polydeoxyribonucleotide) Extraction from Various Plant Species and Its in Vitro Wound Healing Activity. J. Korea Inst. Inf. Electron. Commun. Technol..

[181] Yang C.Y., Han J.S., Lee W.S., Bae J.S., Lee C.W., Jeong E.H., Kim G.H., Park K.H. (2021). The effect of wound healing of *Nile tilapia* (*Oreochromis niloticus*) using PDRN (polydeoxyribonucleotide) extracted from seaweed (*Porphyra* sp.). J. Fish Pathol..

